# KRAS G12D mosaic mutation in a Chinese linear nevus sebaceous syndrome infant

**DOI:** 10.1186/s12881-015-0247-1

**Published:** 2015-10-31

**Authors:** Huijun Wang, Yanyan Qian, Bingbing Wu, Ping Zhang, Wenhao Zhou

**Affiliations:** The Molecular Genetic Diagnosis Center, Shanghai Key Lab of Birth Defects, Translational Medicine Research Center of Children’s Development and Disease, Pediatrics Research Institute, Children’s Hospital of Fudan University, Shanghai, 201102 China

**Keywords:** Linear Nevus Sebaceous Syndrome (LNSS), KRAS, Somatic Mosaicism, Epilepsy

## Abstract

**Background:**

Linear nevus sebaceous syndrome (LNSS) is a multisystem disorder that includes nevus sebaceous and central nervous system, ocular and skeletal anomalies. We report the first case of a *KRAS* G12D mosaic mutation in a patient diagnosed with LNSS.

**Case presentation:**

A 3-month-old female with a clinical diagnosis of LNSS presented with intermittent epilepsy. Her mother carefully collected a skin lesion sample from scratched-off scurf obtained from the patient’s nails. DNA was extracted, and long-range PCR was performed to amplify the *KRAS* gene, which was then analyzed by next-generation sequencing. The results revealed the presence of a low-level heterozygous mutation in the *KRAS* gene (c.35C>T; p.G12D, 5 %).

**Conclusions:**

These findings suggest that the *KRAS* somatic mosaic mutation in this patient may have caused her skin and eye lesions and epilepsy. With this correct diagnosis, the infant can be effectively treated.

## Background

Linear nevus sebaceous syndrome (LNSS) is a rare, congenital neurocutaneous syndrome characterized by midline facial skin lesions (linear nevus sebaceous), seizures and mental retardation [1]. Several syndromes, including Schimmelpenning-Feuerstein-Mims (SFM) syndrome, epidermal nevus syndrome, organoid nevus syndrome and Jadassohn nevus phakomatosis (OMIM 163200), have been classified as a group of disorders characterized by various types of epidermal nevi [2–4]. These syndromes occur sporadically with no sexual predilection, and no associated chromosomal abnormalities have been found [3, 5]. Nevus sebaceous (NS), a hallmark of LNSS, is a hamartoma that can be found on the epidermis, hair follicles and sebaceous and apocrine glands [1]. NS is estimated to occur in 0.3 % of newborns. It is characterized by yellowish-orange to pink lesions that are often oval or linear, sharply demarcated and slightly raised and present as waxy or pebbly surfaces on the skin of the head, face and neck. These lesions vary in size from a few millimeters to several centimeters in length. The typical life histories and histopathological findings of patients with sebaceous nevus are age dependent. Approximately 7 % of NS cases may have additional neurological, ocular and skeletal defects. Epilepsy occurs in up to 25 % of patients and is especially common in LNSS, with a reported incidence of as high as 75 % [6].

These disorders are the result of a genetic mosaicism involving a lethal autosomal dominant gene. A case report of discordant monozygotic (MZ) twins with severe SFM has hypothesized that this syndrome may be due to a postzygotic mutation [7, 8]. However, familial aggregation may sometimes be observed. These disorders may be explained by para-dominant inheritance [9]. Recently, somatic mosaicism has been demonstrated with activating RASopathy mutations in *HRAS* or *KRAS* in NS lesional keratinocytes. Mutations can lead to constitutive activation of the RAF-MEK-ERK signaling pathways and result in increased cellular proliferation. The most common mutation is c.37G>C (p.Gly13Arg) in *HRAS*, which is present in >90 % of NS cases [6]. Here, we report a Chinese infant LNSS patient with intermittent epilepsy and ocular pterygium who has a *KRAS* G12D mosaic mutation, which was detected by performing next-generation sequencing on a noninvasively collected skin lesion sample.

## Case presentation

### Clinical characteristics

**Fig. 1:**
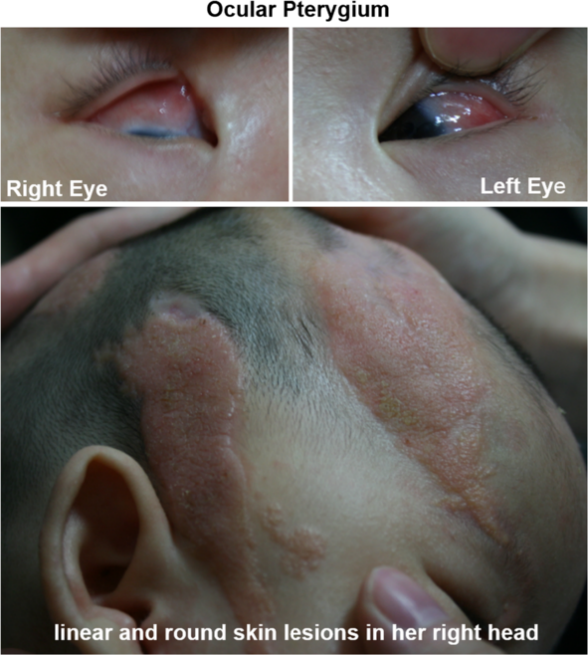
Linear nevus sebaceous involving the skin of the scalp and eyes. There are two linear and one round skin lesion with no hair, present as well demarcated, raised, velvety plaques. The invasive skin lesion near her right eye causes her right eyelid to remain open. She has an ocular pterygium that is pushing down on her right pupil

A female infant was born at 37 + 1 weeks of gestation by cesarean section due to central placenta previa. The young mother experienced polyhydramnios in her last gestation. The birth weight of the baby was 2700 g. Immediately after birth, the infant was found to have multiple skin lesions on her head and eyes (Fig. 1), including two linear and one round skin lesion on the right side of her head. The lesions were well demarcated, raised, velvety plaques containing no hair. An invasive skin lesion was observed on her right eye that caused the eyelid to remain open. She had an ocular pterygium that pushed down on her right pupil. Peripheral blood karyotype analysis revealed a normal female karyotype without any other chromosomal abnormalities. She began to have weak intermittent lip cyanosis at the age of 3 months that lasted up to 3 min and occurred 2–3 times per day. She was a patient at our hospital, and her long-term video EEG showed abnormal waves. Brain CT scan revealed a partially widened gap between the brain and skull, particularly on the right side and the top left side of the temple. No significant changes in the lungs or heart were detected by X-ray or B-ultrasonic examination, respectively. She was diagnosed with new-onset seizures. She is able to grab, use baby talk and listen, but her right eye cannot track her mother’s hand movements. An intelligence test has not been performed. Seizures have been controlled by administration of oxcarbazepine; with this medication, the patient has had fewer seizures.

## Methods

Biopsy of the skin lesion was declined because of seizures. A skin lesion sample from a scratched-off scurf was carefully collected from the patient’s nails and saved in RNA*later*® Stabilization Solution (Thermo Fisher Scientific, USA). Peripheral blood samples were also collected from the patient and her parents. Genomic DNA was extracted from the skin (QIAamp DNA Micro Kit, Germany) and blood samples (QIAamp DNA Blood Mini Kit, Germany). This study was approved by the ethics committee of Children’s Hospital of Fudan University (2014–107) and was performed according to the guidelines of the Declaration of Helsinki.

Targeted next-generation sequencing was performed using an Ion Torrent Personal Genome Machine™ (Life Technology, USA). Both amplicon-based and Ampliseq-based library-building methods were used in this study. Long-range PCR for analyses of the *HRAS* and *KRAS* genes was performed using the patient’s tissue DNA for amplicon-based library building. A PCR product library was prepared according to the instructions provided by the manufacturers of the kits for fragmentation (Ion Shear, Life Technologies, USA) and adaptor and barcode ligation (Ion Xpress Barcode Adapters Kit, Life Technologies, USA). Then, DNA extracted from the patient’s and parents’ blood samples was analyzed with a Noonan Syndrome Ampliseq panel. This panel includes the *NRAS, RIT1, PTPN11, KARS, MAP2K1, SOS1, RAF1* and *BRAF* genes, with coverage of all exons and 10 bp regions from intron/splice sites. The Ampliseq panel library was derived from multiple PCR reactions conducted using Ion AmpliSeq™ HiFi Mix and Ion AmpliSeq™ Primer Pool, in addition to digestion of primer sequences (Ion AmpliSeq™ Library Kits 2.0), and adaptor and barcode ligation (Ion Xpress Barcode Adapters Kit, Life Technologies, USA).

The amplicon- and Ampliseq-based libraries were quantitated using real-time PCR with the Ion TaqMan Assay (Ion Library Quantification Kit, Life Technologies, USA). Construction and enrichment of the emulsion PCR libraries were performed using an Ion OneTouch instrument to clonally amplify the pooled barcoded libraries Ion Sphere™ particles. Sequencing was performed on an Ion 316 sequencing chip according to the manufacturer’s protocol. Torrent Suite™ software was used to compare the base calls. Then, licensed NextGENe software (Softgenetics, USA) was used to complete data analysis. Somatic variants in the skin and blood samples were identified after filtering out germline changes. The allele frequency of a variant was calculated by dividing the number of variant reads by the total number of reads at the same nucleotide position.

## Results

**Fig. 2:**
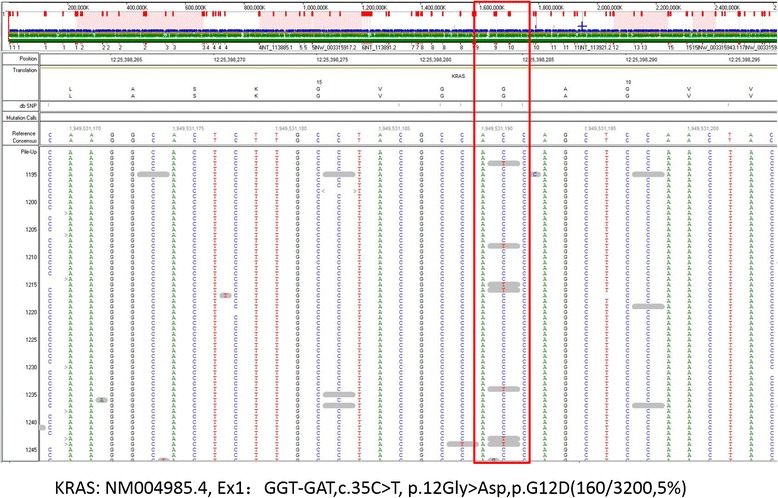
A low-level heterozygous mutation in the *KRAS* gene (c.35C>T; p.G12D, 5 %) was detected in a skin lesion sample collected from a scratched-off scurf

The results showed a mosaic missense mutation in the *KRAS* (NM004985.4) gene in exon 1 (GGT-GAT, c.35C>T, p.12Gly>Asp), resulting in a p.G12D amino acid substitution (Fig. 2). The allele frequency of the altered nucleotide in the skin lesion was 5 % (sequencing depth: 160/3200) in the amplicon-based library. This result was confirmed with the Noonan Syndrome Ampliseq panel (sequencing depth: 21/395). Deep sequencing of DNA extracted from the blood samples from the patient and both parents did not reveal any *KRAS* mutations. No other mutations were detected in the *BRAF* and *HRAS* genes. Sanger-based sequencing was not performed because of the low level of the mosaic mutation.

## Discussion

NS presents at birth, is usually asymptomatic and has a tendency to become more prominent in sebaceous and apocrine components during puberty. Subsequently, secondary tumors, such as syringocystadenoma papilliferum, syringoma, nodular hidradenoma, sebaceous thelioma, and keratoacanthoma, may occur in approximately 25 % of NS cases, though most of them are benign. Because of its cosmetic impact and low but documented malignant potential, prophylactic excision of nevus sebaceous at or before the onset of puberty has been recommended in some studies [4, 10, 11]. However, the recent identification of key epidermal signaling abnormalities that underlie the increased cell proliferation indicates that the development of medical treatments for NS that target the aberrant signaling pathways may be feasible [6].

The *KRAS* gene is located on chr12p12.1 and consists of six exons. Germline mutations in *KRAS* have been identified in patients with cardiofaciocutaneous (CFC) syndrome, Noonan syndrome [12, 13] and Costello syndrome [14]. Somatic *KRAS* mutations frequently occur in lung, colorectal and pancreatic cancers [15, 16]. The somatic *KRAS* c.35G>A mutation is the most frequent *KRAS* mutation in human cancers according to the COSMIC database. However, widespread expression of *KRAS* c.35G>A is not tolerated during mouse embryonic development [17]. Similarly, this mutation has not been observed in human germline cells. Mutations in codons 12, 13 and 61, which are frequent in somatic cancers, have not been identified among germline mutations. Notably, functional analysis has shown that these germline mutations are less activating than the *KRAS* c.35G>A G12D mutation [18, 19].

The first *KRAS* G12D mutation was reported in an infant with epidermal nevus, polycystic kidneys and rhabdomyosarcoma [20]. This mosaic *KRAS* G12D mutation was detected in both the epidermal component of the EN and in the rhabdomyosarcoma, but not in the unaffected tissues or normal skin or blood. This observation strongly suggests a somatic mosaicism. Since the first case, Levinsohn and other authors have identified four additional nevus sebaceous patients with a somatic p.Gly12Asp *KRAS* mutation [21, 22]. These four patients only have nevus sebaceous, with no other characteristics described. Regarding the *KRAS* G12D mutation we report here, the patient was clinically diagnosed with LNSS and displayed linear nevus sebaceous, eye lesions and epilepsy, in contrast with the previously reported NS cases. Our patient had nevus sebaceous with a linear configuration that involved the right side of the face, the scalp and the ocular region, and she also had seizures.

LNSS is difficult to accurately diagnose and has the potential for multisystem organ involvement. Many physicians are unaware of this syndrome, which may delay diagnosis and treatment. In this report, we present an LNSS case and describe the presentation and genetic diagnosis, in addition to incorporating a review of the current literature in this field. Mental retardation affects up to 60 % of LNSS patients and is evident more frequently in patients with hemimegalencephaly [22]. Known cortical abnormalities include focal cortical dysplasia, partial hemimegalencephaly and holohemispheric hemimegalencephaly. A few reports have addressed the utility of cortical resection for epilepsy treatment and have demonstrated significant improvements in seizure frequencies [23, 24].

## Conclusions

In this study, we present the first case of a female infant from China with LNSS and eye lesions. This patent also had a somatic mosaic *KRAS* mutation (c.35G>A; p.Gly12Asp) that was identified in a cutaneous lesion.

## Consent

Written informed consent has been obtained from the parents of the patient.

## Abbreviations

CFC: cardiofaciocutaneous
*KRAS* G12D: *KRAS* gene p.Gly12Asp
LNSS: Linear nevus sebaceous syndrome
OMIM: Online Mendelian Inheritance in Man
SFM: Schimmelpenning-Feuerstein-Mims
